# Smartwatch digital phenotypes predict positive and negative symptom variation in a longitudinal monitoring study of patients with psychotic disorders

**DOI:** 10.3389/fpsyt.2023.1024965

**Published:** 2023-03-13

**Authors:** Emmanouil Kalisperakis, Thomas Karantinos, Marina Lazaridi, Vasiliki Garyfalli, Panagiotis P. Filntisis, Athanasia Zlatintsi, Niki Efthymiou, Asimakis Mantas, Leonidas Mantonakis, Theodoros Mougiakos, Ilias Maglogiannis, Panayotis Tsanakas, Petros Maragos, Nikolaos Smyrnis

**Affiliations:** ^1^Laboratory of Cognitive Neuroscience and Sensorimotor Control, University Mental Health, Neurosciences and Precision Medicine Research Institute “COSTAS STEFANIS”, Athens, Greece; ^2^1st Department of Psychiatry, Eginition Hospital, Medical School, National and Kapodistrian University of Athens, Athens, Greece; ^3^School of Electrical and Computer Engineering (ECE), National Technical University of Athens, Athens, Greece; ^4^Psychiatric Clinic, 414 Military Hospital of Athens, Athens, Greece; ^5^Department of Digital Systems, University of Piraeus, Piraeus, Greece; ^6^2nd Department of Psychiatry, Medical School, University General Hospital “ATTIKON”, National and Kapodistrian University of Athens, Athens, Greece

**Keywords:** schizophrenia, bipolar disorder, relapse prevention, heart rate, heart rate variability, sleep/wake ratio, motion activity

## Abstract

**Introduction:**

Monitoring biometric data using smartwatches (digital phenotypes) provides a novel approach for quantifying behavior in patients with psychiatric disorders. We tested whether such digital phenotypes predict changes in psychopathology of patients with psychotic disorders.

**Methods:**

We continuously monitored digital phenotypes from 35 patients (20 with schizophrenia and 15 with bipolar spectrum disorders) using a commercial smartwatch for a period of up to 14 months. These included 5-min measures of total motor activity from an accelerometer (TMA), average Heart Rate (HRA) and heart rate variability (HRV) from a plethysmography-based sensor, walking activity (WA) measured as number of total steps per day and sleep/wake ratio (SWR). A self-reporting questionnaire (IPAQ) assessed weekly physical activity. After pooling phenotype data, their monthly mean and variance was correlated within each patient with psychopathology scores (PANSS) assessed monthly.

**Results:**

Our results indicate that increased HRA during wakefulness and sleep correlated with increases in positive psychopathology. Besides, decreased HRV and increase in its monthly variance correlated with increases in negative psychopathology. Self-reported physical activity did not correlate with changes in psychopathology. These effects were independent from demographic and clinical variables as well as changes in antipsychotic medication dose.

**Discussion:**

Our findings suggest that distinct digital phenotypes derived passively from a smartwatch can predict variations in positive and negative dimensions of psychopathology of patients with psychotic disorders, over time, providing ground evidence for their potential clinical use.

## 1. Introduction

Schizophrenia and bipolar disorder are the most common psychotic disorders affecting millions worldwide (1). They are chronic disorders and are characterized by repeated relapses after the remission of the first-episode’s symptoms. Relapses lead to worsening of symptoms and progressive cognitive deterioration in schizophrenia (2), cognitive deficits and subsequent functional impairment across all mood states, including periods of remission, in bipolar disorder (3), as well as poorer response to subsequent antipsychotic medication (4) and higher impatient and outpatient costs (5) in both disorders. Therefore, accurate prediction of relapse in psychotic disorders has become a key focus in clinical psychiatry. However, prediction of relapse is highly challenging due to the difficulty of monitoring patients’ symptoms outside the clinic (6).

One promising approach to overcome this issue is digital phenotyping. Recent technological progress in electronic devices miniaturization and multiple physiological data sensors embeddability has allowed for continuous biometric data monitoring using wearable devices such as smartwatches (7). This progress has allowed the application of a novel approach in quantifying human behavior called “digital phenotyping”, using data acquired with wearable devices (8).

Digital phenotyping has been redefined for its application in psychiatry as the moment-by-moment quantification of the individual-level human phenotype in-situ using data from smartphones and other personal digital devices (9). Two distinct categories of these data are active and passive. Active data arise from human-device interaction or activities in which subject’s participation is necessary, such as speech recording in an interview or task, question and answer surveys of behaviors and parameters of the everyday life measure with the device (i.e., smartphone). Collection of passive data does not require the participation of the subject. Recent advancements in technology allow for recording a wide array of passive data including physical activity (*via* accelerometer), heart rate (*via* photoplethysmography), spatial trajectories (*via* GPS), and light exposure (*via* camera) among others (10, 11). Sensor recording from smartphones is considered a more scalable solution due to the wide adoption of these devices (9). However, wearables (fitness trackers and smartwatches) offer the advantages of heart rate measurement and increased reliability in physical activity tracking compared to smartphones (12).

Digital phenotyping techniques have been used to identify novel diagnostic markers in mood disorders (13, 14), suicidality (15), and post-traumatic stress disorder (16). Moreover, recent studies incorporated them for relapse prediction. Barnett et al. (6) collected passive smartphone data for 3 months from seventeen patients with schizophrenia and detected statistically significant anomalies in a 2-week period prior to relapse. Cho et al. (10) collected passive data from smartphone and wearables for 2 years from fifty-five patients with major depressive disorder and bipolar type 1 and 2 disorders, and predicted depressive, manic, and hypomanic episodes in a 3-day period preceding the episode with an accuracy ranging from 87 to 91,2%. Finally, the ongoing multi-center RADAR-MDD study aims to predict major depressive episodes in 600 patients by recording smartphone and wearable passive and active data for a period of 2 years (17).

This study is part of the ongoing e-Prevention project which aims at relapse prediction in patients with psychotic disorders. Digital phenotype data were continuously recorded for a maximum period of 14 months in a group of 35 patients with psychotic disorders (schizophrenia and bipolar disorder) using a commercial smartwatch (Samsung Gear S3, Samsung Group, Republic of Korea). The focus of the current study was to test whether digital phenotypes are related to changes in positive and negative psychopathology of these patients over time (measured using PANSS). We also used a self- reported measure of physical activity (IPAQ) to test if subjectively measured changes in physical activity were related to changes in psychopathology. The goal of this study was to identify objective phenotype measures of clinical significance that could be used as input for modeling relapse prediction.

## 2. Materials and methods

### 2.1. Participants

The sample consists of 35 patients with psychotic disorders (16 patients with Schizophrenia, 2 patients with Schizophreniform Disorder, 2 patients with Schizoaffective Disorder, 13 patients with Bipolar Disorder type I and 2 patients with Bipolar Disorder type II). All patients were sequentially referred by their treating psychiatrists and a diagnostic evaluation was performed at the intake interview by two trained psychiatrists using DSM-5 criteria. Patients were in a remission phase of their psychotic disorder upon entrance to the project.

At the intake interview we recorded demographic data including age, gender, and educational level and screened patients for history of medical, neurological, psychiatric, and developmental disorders, family psychiatric history, birth complications, substance abuse (smoking, alcohol, cannabis, and other psychotropic substances) and Body Mass Index (BMI). We also recorded duration of psychotic disorder before entering the study for each patient. All patients, except three, were receiving antipsychotic medication at entry. In the current analysis the chlorpromazine equivalent dose (18, 19) for each patient for each month of monitoring entered as covariate. We also recorded all other medications including antidepressants, mood stabilizers and anxiolytics for each patient at study entry and subsequently for each month of monitoring (Table 1).

**TABLE 1:** Demographic and clinical characteristics of patients entering the study.

	Schizophrenia spectrum (*n* = 20)	Bipolar spectrum (*n* = 15)	*T*-value (df)	Pearson’s X^2^ (df)	*P*-value
Age	28.8 (7.03)	33.4 (6.75)	–1.95 (33)		0.060
Gender	16 Men, 4 Women	9 Men, 6 Women		1.68 (1)	0.195
Education level	13.05 (1.82)	13.93 (2.71)	–1.15 (33)		0.257
BMI	28.61 (5.43)	26.32 (3.45)	1.43 (33)		0.163
Positive family Psychiatric history	9 (45%)	7 (46.66%)		0.01 (1)	0.922
Disorder duration	5,65 (5.29)	9.6 (7.57)	1.82 (33)		0.078
Smoking	12 (60%)	13 (86.66%)		2.99 (1)	0.084
History of alcohol abuse	6 (30%)	6 (40%)		0.38 (1)	0.537
Chlorpromazine equivalents	662.3 (562.55)	317.33 (257.53)	2.20 (33)		0.035
Antidepressants	8 (40%)	2 (13.33%)		2.99 (1)	0.084
Anxiolytics	0 (0%)	3 (20%)		4.38 (1)	0.036
Mood stabilizers	6 (30%)	13 (86.66%)		8.58 (1)	0.003

Means and standard deviations (parentheses) or number of positive cases and percentages of positive cases (parentheses).

A trained neurologist examined each patient for neurological signs, including motor function, basic mental functions, balance, reflexes, coordination, and gait. Exclusion criteria at the intake interview included age <18 years or >45 years, history of major neurologic and developmental disorders and illegal substance abuse or alcohol abuse within the last year before evaluation.

Total duration of monitoring and data acquisition in the e-Prevention project for each patient will last for 24 months. For the current analysis we retrieved and analyzed data between November 2019 and December 2020 from 35 patients. The minimum time of patients’ data monitoring used in this study was 2 months and the maximum was 14 months.

Researchers gave participants a full description of study design, objective, types of data collected, usage and access of data, as well as expected outcomes. Each participant was given the option to participate either using only the smartwatch for monitoring the basic set of digital phenotypes or using the smartwatch and a tablet (Samsung Galaxy Tab A6) for monitoring the extended set of digital phenotypes (including audiovisual data). Each participant then provided written informed consent for participation in the study and monitoring of the basic or extended set of digital phenotypes. Moreover, participants provided a separate consent form allowing the use of their personal data for the purposes of the e-Prevention research project in compliance with General Data Protection Regulation (GDPR, EU Regulation 2016/679). e-Prevention project study protocol and participant consent forms were reviewed and approved by the Ethics committee of the University Mental Health, Neurosciences and Precision Medicine Research Institute “COSTAS STEFANIS”.

### 2.2. Procedure

#### 2.2.1. Smartwatch day monitoring

Participants received a commercial smartwatch (Samsung Gear S3, Samsung Group, Republic of Korea) and agreed to wear it for the total duration of the study, continuously day and night, excluding charging time and activities that might lead to hardware failure (taking a shower or swimming). Pilot testing showed that this smartwatch required 2 h for charging, thus we aimed to obtain 22 h of useful data within each calendar day for each participant.

Samsung Gear S3 uses the Tizen OS for data collection, but exposes the Tizen API, which can be used to build applications for the smartwatch and access the sensors of the watch in real-time. Using API we created our in-house application, named “e-Prevention”, to continuously collect sensor data without relying on third-party applications. Smartwatch sensors recorded: (1) 3D linear acceleration (accelerometer sensor with gravity compensation), (2) 3D angular velocity (gyroscope sensor), (3) heart measurements *via* photoplethysmography, including beats per minute (BPM) and intervals between successive pulses (PP intervals). The sampling frequency of the smartwatch for raw data was 20 Hz. The kinetic raw data were saved at 20 Hz while the BMP and PP interval data were saved every 200 ms (5 Hz).

Raw data were saved in files in comma-separated format. During charging, data were compressed and transferred *via* WiFi to a cloud server in the “∼okeanos” national public infrastructure-as-a-service. Recordings were organized per user. A randomly generated 24 hexadecimal string was assigned to every user to ensure anonymization. Before data processing, comma-separated files were converted in a column-oriented data storage format (apache parquet) to reduce their size and loading time. More details about system architecture are described in Maglogiannis et al. (20).

#### 2.2.2. Brief interview week monitoring

Participants received a brief interview by one of the research team members, once every week. For participants who gave consent for using the tablet, we used a video call for the interview while for the remaining participants we used telephone call. During interview the participant discussed with research team member about problems with the smartwatch usage and then completed the Greek version of the International Physical Activity Questionnaire–short form (IPAQ-Gr) (21). The questionnaire consists of 7 questions assessing the time spent during the last week on 4 categories of physical activity: vigorous, moderate, walking and sitting. The interviewing researcher recorded IPAQ responses.

#### 2.2.3. Full length interview month monitoring

For the total duration of their participation in the e-Prevention patients agreed to visit the Psychiatry Department of Eginition Hospital once every month for a clinical evaluation during which psychopathology (Positive and Negative Syndrome Scale, PANSS) (22) and Body Mass Index (BMI) were assessed among other clinical parameters. Trained psychiatrists performed all clinical evaluations that did not substitute for the evaluation of each patient by his/her treating psychiatrist. The clinical evaluation of e-Prevention research team did not interfere in any way with the clinical decisions of the treating psychiatrist who was fully responsible for any changes in medication or any other clinical interventions during patient follow up.

### 2.3. Data preprocessing

#### 2.3.1. Smartwatch data

We applied short-time analysis, using non-overlapping time windows, to extract features from the collected data. The analysis window was set to 5 min in accordance with relevant literature on short-time heart rate monitoring (23).

Preprocessing of heart rate data included the calculation of the real heart rate sequence by dropping identical consecutive values and retaining intervals that include at least 90% of valid heart rate data. We replaced PP intervals larger than 2,000 ms and smaller than 300 ms using linear interpolation. Finally, from the preprocessed 5-min interval we retained the first 4.5 min (90%) minutes of PP intervals for feature extraction to mitigate the effect of different percentages of valid measurements across different intervals.

Preprocessing of accelerometer and gyroscope sensor data included replacement of missing values with nearest interpolation, and consideration for feature extraction of intervals with no more than 50 missing values (a 5-min interval has a maximum of 6,000 values). Regarding the PP intervals, we retained the first 5,940 (99%) samples from each preprocessed interval. Wavelet de-noising was applied to mitigate the inherent noise in these sensors’ high frequency (24).

We extracted measures provided by Tizen API and calculated by Samsung’s proprietary algorithms including: (1) awake duration and sleep duration in numbers as well as the ratio of the two (sleep/wake ratio), (2) number of steps covered during wake hours (walking, running and total).

#### 2.3.2. IPAQ questionnaire data

International Physical Activity Questionnaire indicates physical activity (PA) in metabolic equivalents (MET min 1). Here we used the proposed MET estimates of IPAQ which are the following: 8 METs for vigorous PA, 4 METs for moderate PA and 3.3 METs for walking. The total PA is the sum of the times in minutes spent in each PA category multiplied with the values cited for each category.

### 2.4. Data analysis

#### 2.4.1. Derivation of smartwatch phenotypes

The accelerometer and gyroscope 3D signals were used to derive the Short Time Energy of their Euclidean norm over each 5-min time window using the following formula:


$$S\,T\,E=\sum_{j}^{n}x_{j}^{2}$$

where *x* is the norm of the respective accelerometer or gyroscope signal, J is the index of each sample under summation and n is the total number of samples within the time window. The short time energy can be thought of as the traditional measure of total signal energy (i.e., the sum of the squared signal values), which can intuitively be seen as a quantitative measure of activity across the window which is used for summing. This is close to the interpretation of the activity counts of an ActiGraph device (which is a very common metric in the literature), without using thresholding for getting the counts and then summing. This measure was named total motor activity (TMA). For each 5-min interval we derived TMA from the accelerometer and TMA from the gyroscope.

We sued the pulse rate sensor data to derive the Heart Rate Average (HRA) for each 5-min interval. We also used the PP time interval measure from the heart rate sensor to derive standard deviation of PP intervals (SDPP) for each 5-min interval using the formula:


$$S\,D\,P\,P=\sqrt{\frac{1}{n-1}\,\sum_{j=1}^{n}{\left(P\,P_{j}-\bar{P\,P}\right)}^{2}}$$

where $\bar{P\,P}$ is the mean *PP* interval. The SDPP is considered a measure of overall heart rate variability (HRV) (25).

#### 2.4.2. Derivation of API phenotypes

We measured the sleep/wake ratio (SWR) per day for each participant. We also derived the mean number of total steps per minute for each day as a measure of walking activity (WA). To improve the robustness of these measurements we considered data only for days for which we have 18 or more hours recorded.

#### 2.4.3. Derivation of month estimates of digital phenotypes

For each participant, we pooled the mean and standard deviation of the distribution for each one of the smartwatch digital phenotypes as described above, separately for periods of wakefulness and sleep based on the relevant characterization of the smartwatch API over an entire month of monitoring. We did not use the division into wakefulness and sleep aggregates for SWR and WA data. Thus, we derived for each participant for every month the following digital phenotypes:

(a) Mean and standard deviation of accelerometer TMA during wakefulness (wake-TMA), as well as the same values during sleep (sleep-TMA). Preliminary analysis showed that the accelerometer values of mean and SD TMA and the gyroscope values of mean and SD TMA were highly correlated across participants and months both during wakefulness and sleep.
(b) Mean and standard deviation of HRA for each participant and each month during wakefulness (wake-HRA) and sleep (sleep-HRA).
(c) Mean and standard deviation of HRV for each participant and each month during wakefulness (wake-HRV) and sleep (sleep-HRV).
(d) Mean and standard deviation of SWR for each participant and each month.
(e) Mean and standard deviation of WA for each participant and each month.
  In total we derived 16 digital phenotype variables from the smartwatch data for each patient and each month of monitoring.

For each participant and each month of monitoring the number of valid 5-min time windows of motion sensor measurements and the number of valid 5-min time windows of heart rate sensor per month were counted separately during wakefulness and sleep. Months with very low counts (very few days of monitoring) were excluded. The initial sample size was 297 (participant × month) data for wakefulness and 292 (participant × month) data for sleep. We excluded data for total of 13 months from 8 participants resulting in a 284 (participant × month) data set during wakefulness (4.4% excluded data), and a total of 11 months from 8 participants resulting in a 281 (participant × month) data set during sleep (3.8% excluded data).

#### 2.4.4. Derivation of month estimates for physical activity and psychopathology phenotypes

Using the weekly measured data of IPAQ we computed for each participant and each month, the following physical activity (PA) phenotypes: (a) mean Vigorous PA (VPA), mean Moderate PA (MPA), mean walking PA (WPA), and mean total PA (TPA).

We also used PANSS score for each patient for every month to compute 3 psychopathology dimensions: (a) positive psychopathology from the PANSS positive symptom scale score, negative psychopathology from the PANSS negative symptom scale score and general psychopathology from the PANSS general psychopathology symptom scale score.

### 2.5. Statistical analysis

The final data set comprised of month estimates of the 16 smartwatch phenotypes, the 4 physical activity phenotypes and the 3 psychopathology dimensions for each participant. We used Linear mixed effects model analysis to estimate the relation of each smartwatch and physical activity phenotype (independent predictor) on each of the psychopathology dimensions (dependent variable) using SPPS 26 Software (IBM). The smartwatch phenotype was a repeated measures factor in the model using an autoregressive covariance matrix. Each smartwatch and physical activity phenotype entered both as a fixed covariate and as a random covariate with different slope and intercept for each participant. The linear mixed effect model analysis was performed for the set of 16 smartwatch phenotypes for each psychopathology dimension with false discovery rate (FDR) correction of *p*-values for multiple testing (16 tests for each psychopathology dimension). The same analysis was performed for the set of 4 physical activity phenotypes for each psychopathology dimension and again applying FDR correction of *p*-values for multiple testing (4 tests for each psychopathology dimension).

We performed a second set of linear mixed model analyses for those cases were the initial analysis confirmed a significant relation of a phenotype to a psychopathology dimension. We examined the effects of a series of confounding factors on this relation by adding a second fixed factor in the analysis and testing for the interaction of this factor and the phenotype in the prediction of the psychopathology dimension (using *F* tests). We thus performed 10 analyses, one for each factor. The first four factors were age, gender, education level and BMI. We used age and education level to group our participants. Two age groups with similar number of observations, a group of young participants (18–30 year) and a group of older participants (31–45 year), and two groups of similar number of observations, a low education group (12 years of formal education) and a high education group (above 12 years of formal education). We used BMI measures for each participant to form three groups with similar number of observations: normal weight: BMI < 25, overweight: BMI > = 25 and BMI < = 30, obese: BMI > 30).

The next three factors involved clinical characteristics. The first factor grouped patients in the schizophrenia spectrum (schizophrenia and schizoaffective disorder) and the bipolar spectrum (bipolar I and bipolar II). The second grouping factor was chronic disorder (>5 years duration) and recent onset (< = 5 years of duration). The third grouping factor was positive family history for a psychiatric disorder and negative family history.

The final set of factors was related to use of psychotropic agents. One factor was antipsychotic medication dose transformed into chlorpromazine equivalents that was a continuous covariate and not a fixed factor because it could vary for each patient and each month. We did not include a factor for benzodiazepine use since only 3 patients received benzodiazepines, neither a factor for antidepressants nor mood stabilizers because their use was strongly dependent on the diagnosis of bipolar disorder. Another factor grouped participants into smokers and non-smokers and the final factor grouped participants into those drinking alcohol (but not abusing alcohol since this was an exclusion criterion) and those not drinking alcohol. *P*-values were FDR corrected for multiple testing (10 tests for each digital phenotype).

## 3. Results

### 3.1. Relation of phenotypes to psychopathology

All correlations among the same phenotypes measured during sleep and during wakefulness were small to modest (Supplementary Table 1). The largest correlations were between mean and standard deviation of the same phenotype and between mean wake-TMA and mean WA as expected from the fact that the API uses the accelerometer data to measure steps per minute. Correlations among digital phenotypes and physical activity phenotypes were small (Supplementary Table 2).

**FIGURE 1:**
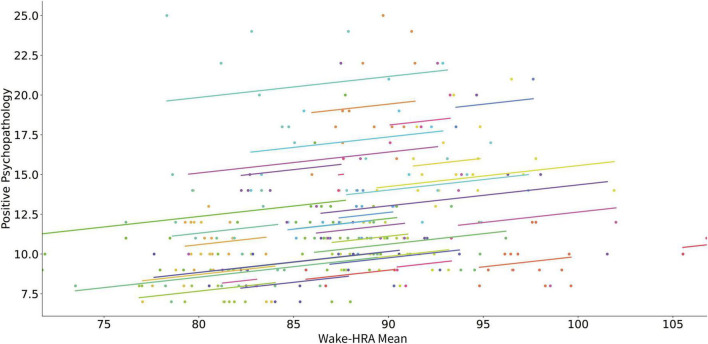
Scatter plot of the relation of mean HRA during wakefulness and positive psychopathology (positive PANSS score). Each subject’s data as well as the predicted linear model line are presented with a different color.

**FIGURE 2:**
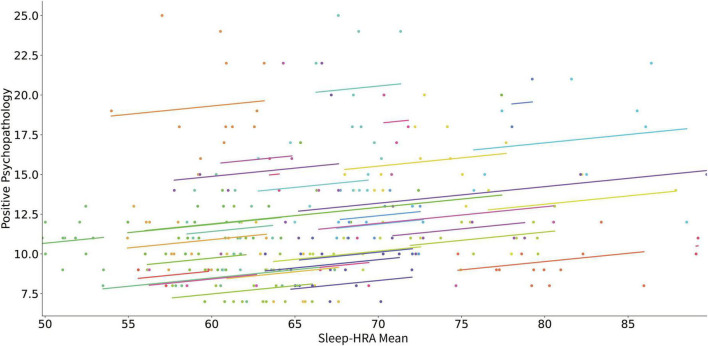
Scatter plot of the relation of mean HRA during sleep and positive psychopathology (positive PANSS score). Each subject’s data as well as the predicted linear model line are presented with a different color.

Table 2 presents the results of linear mixed model analyses using digital phenotypes to predict changes in positive PANSS score for all patients. An increase of the mean wake-HRA (Figure 1) and sleep-HRA (Figure 2) was related to an increase in positive PANSS score. Also, a decrease of wake-HRA standard deviation for each month predicted an increase in positive PANSS score but this effect did not survive correction for multiple testing. Finally, mean, and standard deviation of SWR for each month decreased with increasing positive PANSS score (less sleep related to larger score) but again these effects did not survive correction for multiple testing. All other digital phenotypes were not significantly related to the positive PANSS score.

**TABLE 2:** Digital phenotypes predicting positive psychopathology.

Phenotypes	Estimate (SE)	F, *t*-value (df)	*P*-value
**Total motor activity (accelerometer)**			
Wake-TMA mean	0.26 (0.17)	2.29, 1.49 (95)	0.134
Wake-TMA SD	–0.07 (0.17)	0.16, –0.40 (113)	0.690
Sleep-TMA mean	–1.68 (1.58)	1.12, –1.06 (146)	0.291
Sleep-TMA SD	–0.27 (0.71)	0.11, –0.38 (83)	0.704
**Heart rate average**			
Wake-HRA mean	0.15 (0.05)	10.7, 3.27 (150)	0.001*
Wake-HRA SD	–0.25 (0.1)	5.82, –2.41 (144)	0.017
Sleep-HRA mean	0.11 (0.04)	8.19, 2.86 (208)	0.005*
Sleep-HRA SD	–0.09 (0.11)	0.63, –0.79 (130)	0.430
**Heart rate variability**			
Wake-HRV mean	0.01 (0.01)	1.27, 1.13 (223)	0.260
Wake-HRV SD	–0.03 (0.03)	1.43, –1.19 (224)	0.233
Sleep-HRV mean	–0.02 (0.01)	1.32, –1.15 (144)	0.252
Sleep-HRV SD	–0.02 (0.02)	1.94, –1.02 (192)	0.309
**Walking activity (steps per minute)**			
WA mean	0.0001 (0.0002)	0.23, 0.47 (60)	0.636
WA SD	0.0002 (0.0003)	0.27, 0.52 (58)	0.602
**Sleep wake ratio**			
SWR mean	–2.27 (0.86)	6.92, –2.63 (83)	0.010
SWR SD	–2.65 (1.03)	6.56, –2.56 (68)	0.013

The estimate of the fixed effect of each phenotype predicting the positive dimension of psychopathology (PANSS positive symptom scale score) is presented; SE, standard error of estimate.

*FDR corrected *p* < 0.05.

**FIGURE 3:**
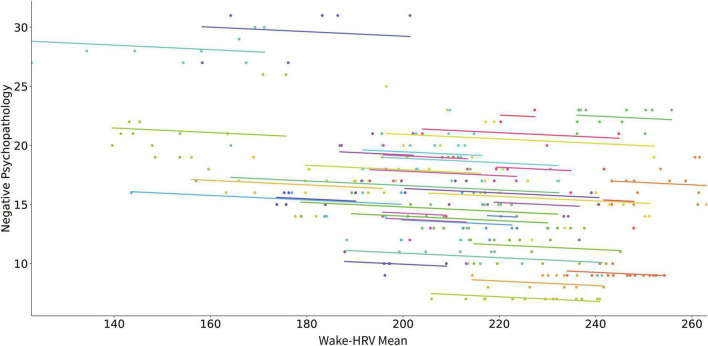
Scatter plot of the relation of mean HRV during wakefulness and negative psychopathology (negative PANSS score). Each subject’s data as well as the predicted linear model line are presented with a different color.

**FIGURE 4:**
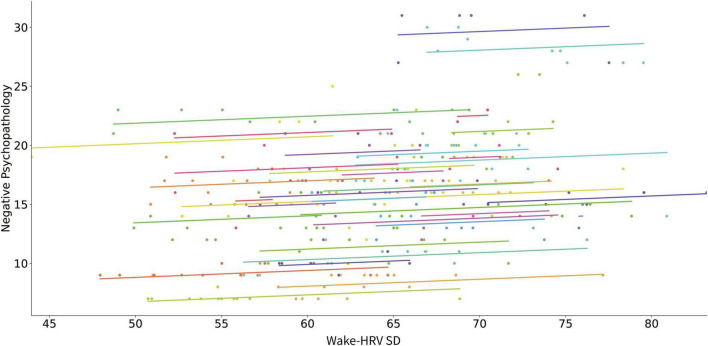
Scatter plot of the relation of the SD of HRV during wakefulness and negative psychopathology (negative PANSS score). Each subject’s data as well as the predicted linear model line are presented with a different color.

**FIGURE 5:**
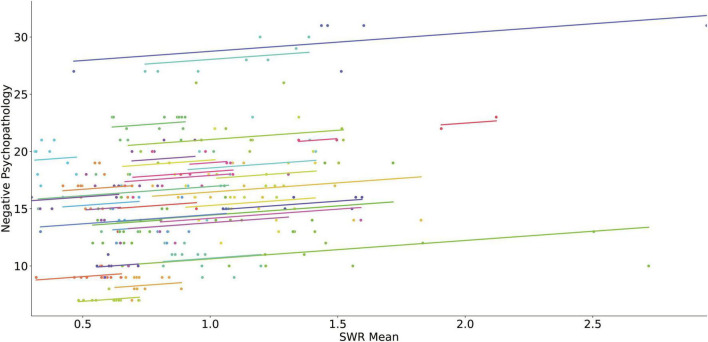
Scatter plot of the relation of SWR and negative psychopathology (negative PANSS score). Each subject’s data as well as the predicted linear model line are presented with a different color.

Table 3 presents the results of the linear mixed model analyses using digital phenotypes to predict changes in negative PANSS score for all patients. An increase in mean of wake-HRV for each month predicted a decrease in negative PANSS score (Figure 3). The increase in standard deviation of wake-HRV for each month predicted an increase in negative PANSS score (Figure 4). Finally, an increase in mean SWR predicted an increase in negative PANSS score (Figure 5). All other digital phenotypes were not significantly related to negative PANSS score.

**TABLE 3:** Digital phenotypes predicting negative psychopathology.

Phenotypes	Estimate (SE)	F, *t*-value (df)	*P*-value
**Total motor activity (accelerometer)**			
Wake-TMA mean	–0.33 (0.25)	1.77, –1.33 (29)	0.193
Wake-TMA SD	–0.23 (0.19)	1.53, –1.24 (58)	0.221
Sleep-TMA mean	2.30 (1.62)	2.01, 1.42 (152)	0.158
Sleep-TMA SD	0.05 (0.84)	0.003, 0.06 (83)	0.954
**Heart rate average**			
Wake-HRA mean	0.002 (0.04)	0.002, 0.05 (226)	0.963
Wake-HRA SD	–0.18 (0.12)	2.23, –1.49 (122)	0.138
Sleep-HRA mean	0.03 (0.03)	0.73, 0.85 (110)	0.396
Sleep-HRA SD	0.02 (0.13)	0.02, 0.16 (81)	0.875
**Heart rate variability**			
Wake-HRV mean	–0.02 (0.01)	7.25, –2.69 (183)	0.008*
Wake-HRV SD	0.07 (0.03)	7.91, 2.81 (225)	0.005*
Sleep-HRV mean	0.01 (0.02)	0.74, 0.86 (111)	0.393
Sleep-HRV SD	0.02 (0.02)	0.55, 0.74 (126)	0.459
**Walking activity (steps per minute)**			
WA mean	0.0001 (0.41)	0.00, 0.00 (20)	1
WA SD	0.0004 (0.48)	0.00, 0.001 (29)	0.999
**Sleep wake ratio**			
SWR mean	2.53 (0.92)	7.5, 2.74 (91)	0.007*
SWR SD	1.48 (1.12)	1.74, 1.32 (86)	0.191

The estimate of the fixed effect of each phenotype predicting the negative dimension of psychopathology (PANSS negative symptom scale score) is presented; SE, standard error of estimate.

*FDR corrected *p* < 0.05.

There were no significant effects of digital phenotypes on the general psychopathology PANSS score (Supplementary Table 3). There were no significant effects of the physical activity phenotypes measured with IPAQ to all dimensions of psychopathology, positive, negative, and general psychopathology (Supplementary Tables 4–6).

### 3.2. Modulation by demographic, clinical, and pharmacological factors

Mean wake-HRA and mean sleep HRA remained significant predictors of positive PANSS score after covariation with all confounding factors (Table 4). There was one significant interaction of mean wake-HRA with family history at predicting positive PANSS score. The positive effect of mean wake-HRA on positive PANSS score was present only for patients with a positive family history of psychiatric disorders (estimate 0.3; SE: 0.06) while for patients with negative family history resulted in a decrease of this estimate by –0.31 (SE: 0.09) resulting in no effect of mean wake-HRA on positive PANSS score.

**TABLE 4:** Digital phenotype interactions with demographic, clinical and pharmacological factors in predicting positive psychopathology.

Confounding factors	Phenotype F(p)	Factor F(p)	Interaction F(p)
**Wake-HRA mean**			
Age	8.63 (0.004)*	1.81 (0.181)	1.70 (0.194)
Gender	7.78 (0.006)*	0.13 (0.718)	0.33 (0.568)
Education	9.77 (0.002)*	1.27 (0.261)	0.92 (0.338)
BMI	8.51 (0.004)*	1.43 (0.242)	1.45 (0.238)
Diagnosis	9.10 (0.003)*	6.30 (0.013)	5.50 (0.020)
Duration	11.23 (0.001)*	0.10 (0.909)	0.13 (0.721)
Family history	10.19 (0.002)*	12.07 (0.001)*	12.35 (0.001)*
Antipsychotic medication	15.00 (0.0002)*	2.97 (0.089)	1.26 (0.264)
Smoking	7.00 (0.009)*	2.21 (0.139)	2.11 (0.148)
Alcohol	8.34 (0.004)*	0.03 (0.857)	0.02 (0.886)
**Sleep-HRA mean**			
Age	5.88 (0.016)*	0.29 (0.590)	0.20 (0.655)
Gender	4.73 (0.031)*	2.51 (0.115)	3.85 (0.051)
Education	6.41 (0.012)*	2.15 (0.144)	1.23 (0.269)
BMI	5.35 (0.022)*	2.21 (0.112)	1.91 (0.151)
Diagnosis	6.86 (0.010)*	4.03 (0.046)	2.85 (0.093)
Duration	5.93 (0.016)*	0.58 (0.446)	1.05 (0.306)
Family history	7.05 (0.009)*	0.34 (0.561)	0.39 (0.534)
Antipsychotic medication	0.69 (0.021)*	0.08 (0.93)	0.37 (0.546)
Smoking	11.22 (0.001)*	4.49 (0.035)	4.23 (0.041)
Alcohol	4.42 (0.037)*	0.77 (0.380)	0.83 (0.364)

*F*-value of the fixed effect for the digital phenotype (presented in column 1 with bold characters) is shown in column 2, *F*-value of the fixed effect for each confounding factor (presented in column 1) is shown in column 3 and *F*-value for the interaction of digital phenotype and factor is shown in column 4. *P*-values are shown in parentheses.

*FDR corrected *p* < 0.05.

Standard deviation of wake-HRV remained significant predictor of negative PANSS score after covariation with all confounding factors (Table 5) while mean HRV did not remain significant after covariation with BMI and SWR did not remain significant after covariation with gender (Table 5). There was one significant interaction between antipsychotic medication and wake-HRV standard deviation in predicting negative PANSS score. Both increases in wake-HRV standard deviation (estimate: –0.18, SE:0.036) and increases in antipsychotic medication (estimate: –0.01, SE:0.002) were related to decrease in negative PANSS score and these effects interacted negatively with each other (interaction estimate: –0.00002, SE: 0.00006).

**TABLE 5:** Digital phenotype interactions with demographic, clinical and pharmacological factors in predicting negative psychopathology.

Confounding factors	Phenotype F(p)	Factor F(p)	Interaction F(p)
**Wake-HRV mean**			
Age	6.97 (0.009)*	2.45 (0.119)	0.37 (0.542)
Gender	8.57 (0.004)*	0.08 (0.775)	1.97 (0.161)
Education	8.50 (0.004)*	1.05 (0.306)	3.06 (0.081)
BMI	3.86 (0.051)	1.73 (0.180)	2.22 (0.111)
Diagnosis	8.41 (0.004)*	0.72 (0.398)	0.04 (0.836)
Duration	7.15 (0.008)*	0.07 (0.796)	0.01 (0.918)
Family history	7.36 (0.007)*	0.003 (0.955)	0.32 (0.569)
Antipsychotic medication	23.58 (0.00002)*	6.25 (0.013)	2.64 (0.107)
Smoking	5.90 (0.017)*	0.40 (0.527)	0.26 (0.611)
Alcohol	9.32 (0.003)*	0.98 (0.322)	2.74 (0.099)
**Wake-HRV SD**			
Age	7.29 (0.007)*	0.23 (0.632)	0.09 (0.770)
Gender	7.90 (0.005)*	5.06 (0.025)	0.33 (0.564)
Education	5.31 (0.022)*	0.52 (0.471)	0.09 (0.768)
BMI	5.98 (0.015)*	3.50 (0.032)	1.59 (0.206)
Diagnosis	12.70 (0.0004)*	8.66 (0.004)*	2.95 (0.087)
Duration	8.11 (0.005)*	1.32 (0.251)	1.19 (0.277)
Family history	7.72 (0.006)*	2.15 (0.144)	0.69 (0.406)
Antipsychotic medication	25.6 (0.0000008)*	17.37 (0.00005)*	8.6 (0.004)*
Smoking	4.95 (0.027)*	4.77 (0.030)	3.62 (0.058)
Alcohol	9.49 (0.002)*	5.09 (0.025)	1.60 (0.208)
**SWR mean**			
Age	7.15 (0.009)*	1.08 (0.302)	0.16 (0.686)
Gender	2.50 (0.117)	2.12 (0.149)	1.26 (0.265)
Education	6.11 (0.015)*	2.68 (0.106)	0.03 (0.868)
BMI	8.42 (0.005)*	3.22 (0.045)	1.21 (0.304)
Diagnosis	7.40 (0.008)*	3.94 (0.050)	0.23 (0.635)
Duration	7.42 (0.008)*	0.20 (0.875)	0.11 (0.738)
Family history	7.03 (0.009)*	1.15 (0.286)	0.14 (0.707)
Antipsychotic medication	7.34 (0.008)*	0.27 (0.603)	0.85 (0.359)
Smoking	6.03 (0.016)*	0.14 (0.707)	0.15 (0.698)
Alcohol	4.81 (0.031)*	0.01 (0.901)	1.75 (0.189)

*F*-value of the fixed effect for the digital phenotype (presented in column 1 with bold characters) is shown in column 2, *F*-value of the fixed effect for each confounding factor (presented in column 1) is shown in column 3 and *F*-value for the interaction of digital phenotype and factor is shown in column 4. *P*-values are shown in parentheses.

*FDR corrected *p* < 0.05.

## 4. Discussion

This study used digital phenotypes to predict changes in psychopathology in outpatients with schizophrenia and bipolar disorder. Digital phenotypes were continuously recorded with a smartwatch over a period of up to 14 months. Psychopathology was assessed using PANSS in monthly intervals. Increased average heart rate (HRA) during wakefulness and sleep correlated with increased positive psychopathology. Besides, decreased heart rate variability (HRV) and increased HRV monthly variability during wakefulness correlated with increased negative psychopathology.

In line with previous studies that have found a relation between increased HRA and positive psychopathology (26–29), our study confirms this relation in long term monitoring of patients. It has been suggested that greater psychotic symptom severity is associated with patients’ poorer autonomic functioning indexed with increased heart rate among other autonomic features, as a stress response to changing environmental demands (30, 31). Nevertheless, an opposite relation of HRA and positive psychopathology was reported in one study, in which BPRS was used instead of PANSS (30).

The relation of heart rate and positive psychopathology remained significant after covariation with confounding factors including demographic (age, gender, education, BMI), clinical (diagnosis, family history for psychiatric disorder, years of illness), and pharmacological (antipsychotic medication, alcohol use and smoking) factors. Again, our findings are in line with studies reporting increased heart rate in drug naïve patients at first episode of psychosis and unmedicated patients with positive psychopathology (26, 32).

Moreover, we found that the relation of increased monthly HRA during wakefulness and positive psychopathology was stronger for patients with a positive family history. Whereas the relation weakened for patients with a negative family history. Given that patients with a positive family history of any psychiatric disorder present more severe positive and emotional symptoms measured with PANSS (33), our finding suggests that genotype may somehow modulate the relation between heart rate digital phenotypes and psychopathology.

The negative symptom domain of PANSS, which largely focuses on distortions in emotional, social, and thinking processes, has been inversely related to HRV parameters in groups of patients with schizophrenia (28, 31, 34). Our study confirms this relation in long term monitoring.

The relation of mean HRV and its monthly variance with negative psychopathology remained significant after covariation with all confounding factors except for BMI, which nullified the relation of mean HRV and negative psychopathology. Besides, antipsychotic medication modulated the relation between monthly HRV variance and negative psychopathology.

Finally, we found that an increase in mean sleep wake ratio correlated with increased negative psychopathology. This relation remained significant after covariation with all confounding factors except gender. An increase in sleep duration is a symptom of a circadian rhythm disruption and is related to increase of negative psychopathology. However, a common characteristic of patients with negative psychopathology is that they tend to stay in bed for long hours doing nothing, not necessarily sleeping. Since the smartwatch cannot really differentiate this state of reduced activity from sleep, one should be cautious interpreting the increase of sleep measured using the smartwatch as an actual disruption in sleep pattern.

In our study we did not find any relation of subjectively measured physical activity (IPAQ) with changes in psychopathology. We consider this an important negative finding since weekly measured physical activity with a simple questionnaire could be an easy to implement alternative to digital phenotypes if a relation to psychopathology could be detected. The fact that no such relation could be detected in our sample suggests that self-reported physical activity cannot substitute for digital phenotypes.

Summarizing, our results are part of the ongoing e-Prevention project and indicate that heart rate and sleep wake ratio phenotypes successfully predicted changes in positive and negative psychopathology, while motion related digital phenotypes (TMA and WA) and subjectively measured physical activity (IPAQ) did not. These results are important for guiding our investigation of digital phenotypes as potential biomarkers in the prediction of psychotic relapse in these patients and suggest that specifically phenotypes associated with cardiac function (HRA, HRV) and circadian rhythm (SWR) are promising candidates. Different patterns of relations emerged for positive and negative psychopathology suggesting that digital phenotypes can also dissociate positive and negative symptom increase. Also, there was no relation between digital phenotypes and measures of general psychopathology, thus highlighting the specificity of the relation that we found between distinct psychopathological dimensions and different digital phenotypes. Finally, the relation to specific digital phenotypes with psychopathology was similar for patients with schizophrenia and patients with bipolar disorder. This is also a very important finding guiding our further analysis in the e-prevention project suggesting that the same digital phenotypes could potentially be used for psychotic relapse prediction in both disorders.

The strength of this study was the continuous longitudinal recording of digital phenotypes as well as the long term recording of several clinical variables for each patient including assessment of psychopathology and medication status. Thus, although the sample size of 35 patients was medium the overall data set for these patients was much larger. One the other hand a limitation of the study was the small sample size of participants for each diagnostic group. Another limitation was the collapse of individual digital phenotype data over a month period to compare them with psychopathology data, resulting in the loss of fine time structure information. Given that data collection continues currently for most patients, future analyses on e-Prevention data will use more refined analysis tools to include all time points of individual digital phenotype data.

In conclusion this study confirmed that specific digital phenotypes derived from a smartwatch can predict within subject variations in positive and negative dimensions of psychopathology over time in a sample of patients with psychotic disorders (schizophrenia and bipolar disorder). These preliminary results provide proof of concept for the ongoing e-Prevention project. This project uses continuous (24 h) monitoring of digital phenotypes for a period of 12–24 months and detailed monthly clinical evaluation for every patient aiming to predict psychotic symptom relapse. The next step is to use automated algorithms using machine learning modeling for effective monitoring and relapse prediction and prevention in psychotic disorders.

## Data availability statement

The raw data supporting the conclusions of this article will be made available by the authors, without undue reservation.

## Ethics statement

The e-Prevention project study protocol and participant consent forms were reviewed and approved by the Ethics Committee of the University Mental Health, Neurosciences and Precision Medicine Research Institute “COSTAS STEFANIS”. The patients/participants provided their written informed consent to participate in this study. Written informed consent was obtained from the individual(s) for the publication of any potentially identifiable images or data included in this article. The participants provided a separate consent form allowing the use of their personal data for the purposes of the e-Prevention research project in compliance with General Data Protection Regulation (GDPR, EU Regulation 2016/679).

## Author contributions

EK participated in study design, patient assessment and smartwatch data collection, data analysis, and writing of the manuscript. TK participated in study design. smartwatch data collection, data analysis, and writing of the manuscript. ML participated in study design, patient assessment, data analysis, and writing of the manuscript. VG participated in patient assessment and analysis. PF, AZ, and NE participated in study design, smartwatch data pre-processing, and analysis. AM, LM, and TM participated in patient assessment and analysis. IM, PT, and PF participated in study design, smartwatch data pre-processing, data analysis, and editing of the manuscript. All authors contributed to the article and approved the submitted version.
