# Supplementary material for: Smartwatch digital phenotypes predict positive and negative symptom variation in a longitudinal monitoring study of patients with psychotic disorders
Source: Front Psychiatry. 2023 Mar 13;14:1024965. doi: 10.3389/fpsyt.2023.1024965 (PMC10040533; doi:10.3389/fpsyt.2023.1024965)
Supplement: Supplementary file 1 [file Table_1.DOCX]

**Supplementary Table 1: Correlations (Pearson) among smartwatch digital phenotypes**

|  | wake-TMA Mean | wake-TMA SD | sleep-TMA Mean | sleep-TMA SD | wake-HRA Mean | wake-HRA SD | sleep-HRA Mean | sleep-HRA SD | wake-HRV Mean | wake-HRV SD | sleep-HRV Mean | sleep-HRV SD | WA Mean | WA SD | SWR Mean |
| --- | --- | --- | --- | --- | --- | --- | --- | --- | --- | --- | --- | --- | --- | --- | --- |
| **Total Motor Activity (Accelerometer)** | | | | | | | | | | | | | | | |
| wake-TMA Mean |  |  |  |  |  |  |  |  |  |  |  |  |  |  |  |
| wake-TMA SD | .65 |  |  |  |  |  |  |  |  |  |  |  |  |  |  |
| sleep-TMA Mean | .09 | .26 |  |  |  |  |  |  |  |  |  |  |  |  |  |
| sleep-TMA SD | .07 | .25 | .89 |  |  |  |  |  |  |  |  |  |  |  |  |
| **Heart Rate Average** | | | | | | | | | | | | | | | |
| wake-HRA Mean | .30 | .05 | .24 | .21 |  |  |  |  |  |  |  |  |  |  |  |
| wake-HRA SD | .20 | .40 | .00 | -.13 | -.02 |  |  |  |  |  |  |  |  |  |  |
| sleep-HRA Mean | -.22 | -.17 | .43 | .47 | .55 | -.48 |  |  |  |  |  |  |  |  |  |
| sleep-HRA SD | .06 | .27 | .35 | .25 | .25 | .63 | .10 |  |  |  |  |  |  |  |  |
| **Heart Rate Variability** | | | | | | | | | | | | | | | |
| wake-HRV Mean | .60 | .27 | -.06 | -.04 | .43 | .06 | .02 | 07 |  |  |  |  |  |  |  |
| wake-HRV SD | -.43 | -.06 | -.03 | .03 | -.35 | .08 | -.01 | 03 | -.65 |  |  |  |  |  |  |
| sleep-HRV Mean | .36 | .34 | .14 | .05 | .14 | .55 | -.29 | .58 | .46 | -.33 |  |  |  |  |  |
| sleep-HRV SD | .22 | .16 | .09 | .04 | .30 | .43 | .00 | .50 | .27 | -.10 | .57 |  |  |  |  |
| **Walking Activity (steps per minute)** | | | | | | | | | | | | | | | |
| WA Mean | .79 | .47 | -.08 | -0.12 | 0.25 | .42 | -.41 | .14 | .44 | -.31 | .38 | .41 |  |  |  |
| WA SD | .45 | .43 | -.08 | -0.11 | 0.02 | .50 | -.42 | .16 | .19 | -.05 | .31 | .27 | .67 |  |  |
| **Sleep Wake Ratio** | | | | | | | | | | | | | | | |
| SWR Mean | -.40 | -.15 | -.21 | -0.22 | -0.12 | .20 | -.13 | .04 | -.29 | .44 | -.09 | .04 | -.19 | -.01 |  |
| SWR SD | -.30 | -.08 | -.13 | -0.15 | -0.17 | .18 | -.09 | .11 | -.26 | .40 | -.05 | .05 | -.16 | .03 | .76 |

**Supplementary Table 2: Correlations (Pearson) of smartwatch digital phenotypes with subjective physical activity phenotypes (IPAQ)**

|  | VPA | MPA | WPA | TPA |
| --- | --- | --- | --- | --- |
| **Total Motor Activity (Accelerometer)** | | | | |
| wake-TMA Mean | .11 | .26 | .27 | .34 |
| wake-TMA SD | .15 | .07 | .27 | .27 |
| sleep-TMA Mean | -.19 | .08 | .06 | -.02 |
| sleep-TMA SD | -.13 | .14 | .06 | .03 |
| **Heart Rate Average** | | | | |
| wake-HRA Mean | -.09 | .11 | .22 | .15 |
| wake-HRA SD | .01 | -.09 | .22 | .10 |
| sleep-HRA Mean | -.10 | .07 | -.08 | -.06 |
| sleep-HRA SD | -.13 | -.02 | .24 | .07 |
| **Heart Rate Variability** | | | | |
| wake-HRV Mean | .11 | .29 | .23 | .33 |
| wake-HRV SD | -.04 | -.20 | -.10 | -.18 |
| sleep-HRV Mean | -.07 | -.04 | .16 | .05 |
| sleep-HRV SD | -.08 | -.02 | .19 | .06 |
| **Walking Activity (steps per minute)** | | | | |
| WA Mean | .11 | .19 | .41 | .39 |
| WA SD | .25 | .08 | .31 | .35 |
| **Sleep Wake Ratio** | | | | |
| SWR Mean | .00 | -.27 | .00 | -.13 |
| SWR SD | -.07 | -.19 | -.05 | -.15 |

**Note:** VPA: Vigorous Physical Activity, MPA: Moderate Physical Activity, WPA: Walking Physical Activity, TPA: Total Physical Activity.

**Supplementary Table 3: Digital phenotypes predicting general psychopathology**

| Phenotypes | Estimate (SE) | F, t value (df) | p value |
| --- | --- | --- | --- |
| **Total Motor Activity (Accelerometer)** | | | |
| wake-TMA Mean | -.52(.27) | 3.84, -1.96(102) | .053 |
| wake-TMA SD | -.40(.23) | 2.99, -1.73(122) | .086 |
| sleep-TMA Mean | -.17(2.48) | .005, -.07(145) | .944 |
| sleep-TMA SD | -.28(1.11) | .07, -.26(78) | .799 |
| **Heart Rate Average** | | | |
| wake-HRA Mean | .13(.07) | 3.77, 1.94(203) | .054 |
| wake-HRA SD | -.05(.17) | .08, -.28(134) | .783 |
| sleep-HRA Mean | .09(.06) | 2.4, 1.55(190) | .123 |
| sleep-HRA SD | .20(.18) | 1.18, 1.08(131) | .280 |
| **Heart Rate Variability** | | | |
| wake-HRV Mean | -.02(.02) | 2.29, -1.51(192) | .132 |
| wake-HRV SD | .08(.04) | 3.1, 1.76(243) | .079 |
| sleep-HRV Mean | -.002(.02) | .01, -.11(140) | .910 |
| sleep-HRV SD | -.02(.03) | .25, -.50(196) | .616 |
| **Walking Activity (steps per minute)** | | | |
| WA Mean | -.0001(.0003) | .18, -.43(54) | .672 |
| WA SD | .0002(.0005) | .21, .46(52) | .649 |
| **Sleep Wake Ratio** | | | |
| SWR Mean | 1.21(1.32) | .84, .92(110) | .362 |
| SWR SD | -.25(1.56) | .03, -.16(84) | .872 |

**Note:** the estimate of the fixed effect of each phenotype predicting the general psychopathology dimension (PANSS general psychopathology symptom scale score) is presented; SE: standard error of estimate; * FDR corrected p < .05.

**Supplementary Table 4: Physical activity phenotypes predicting positive psychopathology**

| Phenotypes | Estimate (SE) | F, t value (df) | p value |
| --- | --- | --- | --- |
| VPA | .002 (.58) | .00, .004 (39) | .997 |
| MPA | -.02 (.31) | .006, -.07 (19) | .941 |
| WPA | .002 (.002) | .53, .73 (17) | .475 |
| TPA | .002 (.001) | 2.54, 1.59 (24) | .124 |

**Note:** estimate of the fixed effect of each physical activity phenotype (derived from IPAQ) predicting positive psychopathology dimension (PANSS positive psychopathology symptom scale score) VPA: Vigorous Physical Activity, MPA: Moderate Physical Activity, WPA: Walking Physical Activity, TPA: Total Physical Activity. SE: standard error of estimate; *FDR corrected p < 0.05.

**Supplementary Table 5: Physical activity phenotypes predicting negative psychopathology**

| Phenotypes | Estimate (SE) | F, t value (df) | p value |
| --- | --- | --- | --- |
| VPA | -.0002 (.88) | .00 -.00 (49) | 1.0 |
| MPA | .02 (.61) | .002, .04 (55) | .969 |
| WPA | -.002 (.56) | .00, -.003 (49) | .997 |
| TPA | -.001 (.54) | .00, -.002 (46) | .998 |

**Note:** estimate of the fixed effect of each physical activity phenotype (derived from IPAQ) predicting negative psychopathology dimension (PANSS negative psychopathology symptom scale score) VPA: Vigorous Physical Activity, MPA: Moderate Physical Activity, WPA: Walking Physical Activity, TPA: Total Physical Activity. SE: standard error of estimate; *FDR corrected p < 0.05.

**Supplementary Table 6: Physical activity phenotypes predicting general psychopathology**

| Phenotypes | Estimate (SE) | F, t value (df) | p value |
| --- | --- | --- | --- |
| VPA | .01 (.91) | .00, .1 (35) | .990 |
| MPA | -.01 (.5) | .00, -.02 (21) | .985 |
| WPA | .001 (.003) | .18, .42 (21) | .679 |
| TPA | .001 (.002) | .46, .68 (18) | .508 |

**Note:** estimate of the fixed effect of each physical activity phenotype (derived from IPAQ) predicting general psychopathology dimension (PANSS general psychopathology symptom scale score) VPA: Vigorous Physical Activity, MPA: Moderate Physical Activity, WPA: Walking Physical Activity, TPA: Total Physical Activity. SE: standard error of estimate; *FDR corrected p < 0.05.
